# Supplementary material for: Autonomous discovery of optically active chiral inorganic perovskite nanocrystals through an intelligent cloud lab
Source: Nat Commun. 2020 Apr 27;11:2046. doi: 10.1038/s41467-020-15728-5 (PMC7184584; doi:10.1038/s41467-020-15728-5)
Supplement: Supplementary file 1 — Supplementary information [file 41467_2020_15728_MOESM1_ESM.pdf]

Supplementary information for

Autonomous Discovery of Optically Active Chiral Inorganic Perovskite Nanocrystals Through an Intelligent Cloud Lab

By Jiagen Li et al.

Supplementary Method 1.

MAOSIC runs as a web server that receives synthesis request and optimizes the corresponding reaction parameters on the first-in-first-out (FIFO) principle. Supplementary Figure 1 shows the Workflow of how MAOSIC handling the request.

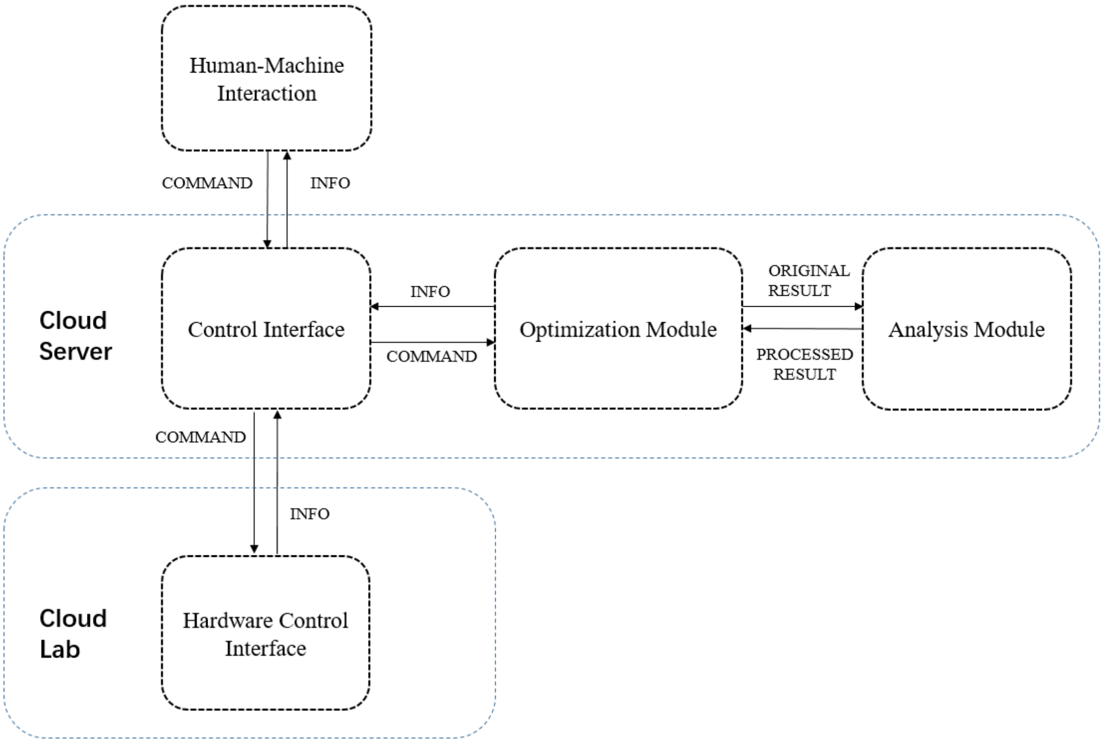

Supplementary Figure 1. Scheme of MAOSIC. Five modules were designed and intercalated with each other for autonomous materials discovery.

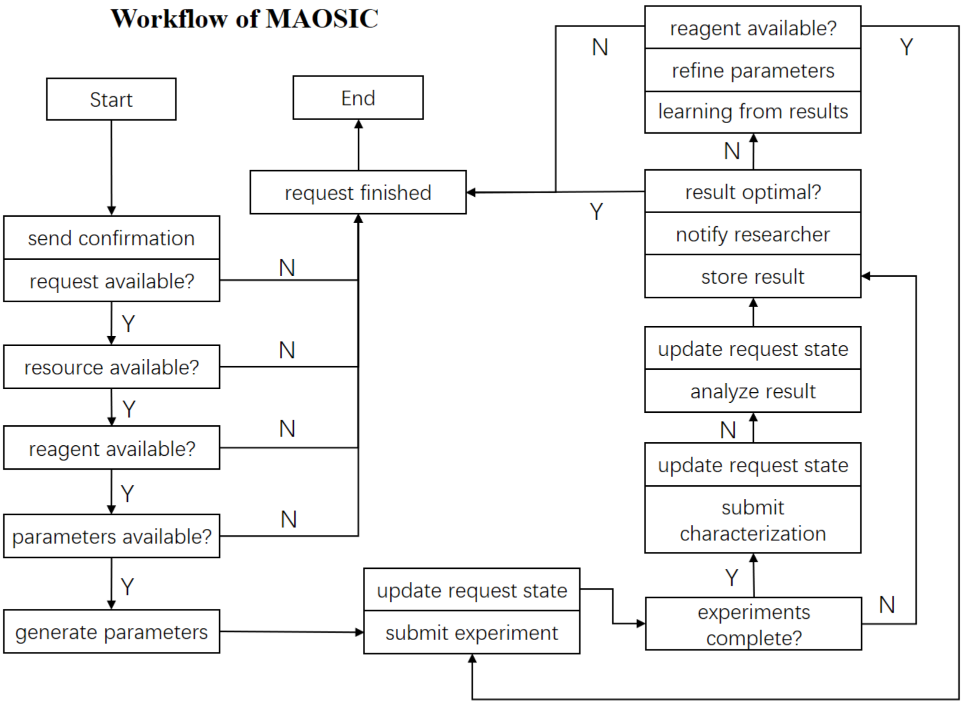

Supplementary Figure 2. The workflow of MAOSIC.

Hardware Requirements: A powerful CPU. Laptop CPUs can often run the software, and the performance will influence software speeds

Hardware: MAOSIC has hardware control interface, which controls all hardware and translates hardware-independent instructions into elementary basic operations.

Without our hardware, MAOSIC cannot communicate with hardware and raise exceptions.

Operating systems: MAOSIC could run on multiple Operating Systems including Windows 10, most Linux based distributions, and MacOS. MAOSIC cores are implemented in Python. So, MAOSIC could run on the most mainstream operating system

Install Guide: Installing dependencies: Python >= 3.5 64-bit

– apt/yum install python3 (Linux)

– Installer (Windows)

– brew install python3 (MacOS)

- Python 3 Anaconda environment is recommended, which could be download from https://www.anaconda.com/download/

MySQL (optional): MAOSIC store long-term information through SQLAlchemy, which support multiple mainstream SQL database. MySQL, Postgres, Oracle, and SQLite are supported in MAOSIC and needs to be configured.

One of the databases must be configured, and MySQL is recommended.

MySQL could be downloaded from https://www.mysql.com/downloads/

Getting the MAOSIC code: MAOSIC can be directly downloaded from GitHub. For now, extract the code to a directory where you are comfortable working with it. Navigate to it with the command line.

Setup: Enter the folder that MAOSIC has been downloaded to and run the command to install Python dependent packages.

```
pip install -r requirements.txt
```

For now, this guide is far from complete. The new functionality may be updated over time, and new dependencies will be added and removed accordingly in the future.

MAOSIC Illustration

MAOSIC has multiple modules. These modules could run on a computer or multiple computers with internet access.

Hardware interface: Control all robotics hardware, handle all hardware dependent information and provide hardware control interface

Arduino: An Arduino Uno board is used to control a stepper motor and two gas valves. MsTimer2 is used to control the time accurately.

STM32: A STM32 F103 development board is used to control the peristaltic pumps and a stepper motor.

PC: Hardware interface communicates with microcontrollers through serial and handles all hardware-dependent information. With our robotics hardware, the hardware interface could work normally. Run `cd hardware_interface/PC & python hardware_control_interface.py`, input the port of each serial and a JSON-RPC based control interface would start on port 8888.

Cloud: Including core of MAOSIC (handle the request and optimize the reaction parameters to meet the demand), a web server for user to submit demand and SQL database (optional).

Three modules (MAOSIC core, a web server and MySQL) could be distributed run on cloud server. MAOSIC core is fully implemented in Python, which could handle the synthesis request, control hardware to do experiments through a hardware control interface, analyze the experiment results, and learn from the results. This module automates the synthesis reaction parameter optimization after a request. The request is implemented base on JSON-RPC protocol.

MAOSIC core includes optimizer and analyzer. For the learning policy of optimizer, an algorithm based on SnobFit is used currently. For analyzer, multi-peak gaussian wave fitting is implemented to analyze the spectrum data.

MAOSIC core needs to fetch and store data from the database management system (DBMS) so that the core could work normally. Run `python run.py` to start MAOSIC core. The request handler would start on port 9999.

A basic version website where the user could submit synthesis demand is implemented in Flask. The web server would start on port 80 after run `cd cloud\website-basic & python run.py`. This website needs to connect the database to store the long-term storage. MAOSIC core should be working when submitting the request.

User control: Provide multiple ways for users to interact with MAOSIC

UI: A simple UI implemented with PyQt is provided in `\user_control\UI`, which could support low-level control of the hardware. Currently, syringe pump control is supported. Open the UI by running `cd user_control\UI & python ui.py`

Browser: Users could connect the website through browser to submit their demands.

Database Config: MAOSIC fetch and store important information from database through SQLAlchemy. So, MAOSIC supports multiple mainstream SQL database such as MySQL, Postgres, Oracle, and SQLite. `maosic\__init__.py` records the database config URI. This database connection URI should be modified (https://flask-sqlalchemy.palletsprojects.com/en/2.x/config/#connection-uri-format) to configure the database.

After configuration, run `cd scripts & python create_db.py` to initialize the database table and insert little test data. Currently, we do not upload all the data in our database.

The database mainly stores the data during experiments. MAOSIC could learn from those data.

It is the first version of MAOSIC; more functionalities are under construction.

Specification of instruction symbols in MAOSIC

All robotic automation and characterization modules are controlled by a hardware interface in MAOSIC, which includes both high-level and low-level instructions based on JSON-RPC2.0 protocol. The high-level instruction is a formula made up of instruction symbols and operators. Each symbol indicates the corresponding hardware module, and each module has its specific parameter vector, which represents all adjustable parameters this module can provide. Taking an example for the *Heating module*, the instruction symbol is H, and the parameter vector is $[T, \dot{T}, t^H]$. Here, T is the stable temperature controlled by H, $\dot{T}$ is the temperature gradient (heating rate), t^H is the working time of H. The symbol array collected by operators can describe the arrangement of modules and reagent in both time and space domain. Five kinds of operators are utilized: $"+"$, $"\times"$, $"\cdot"$ and $"|"$. By combining different module symbols and the operators, a language is developed for the communication between MAOSIC and the real experiments. Details of all instruction symbols, parameter vector (Supplementary Table 1) and operators (Supplementary Table 2) can be found. With the help of the language, all experimental operations can be interpreted by a formula, and then been convert into the machine code, which is defined as process of "compile" (details are shown in supporting information S4, Supplementary Figure 4). All

parameters in an experiment will be stored in vectors to construct the parameter space, which is called "parameterization". These vectors are input data for both hardware control interface and AI optimizer.

Supplementary Table 1. Specification of instruction symbols and parameter vectors in MAOSIC

**Symbol**	**Module**	**Parameters**	**Parameter vector**
H	Heating module	temperature, temperature gradient, time	$[T, \dot{T}, t^H]$
ABS-CD	Abs-CD Measurement	integration time	$[Ip]$
CR	Collaborative robot	initial point, final point, time	$[\mathrm{P}_i, \mathrm{P}_f, \mathrm{tr}]$
SP	Syringe pump	injection volume, time, injection rate, times of injection	$[vi, ti, \eta, \tau]$

Supplementary Table 1 symbolizes and parametrizes all the modules in MAOSIC, with time dependence. We define $\boldsymbol{\theta}$ as the vector that concludes all adjustable parameters as:

$$\boldsymbol{\theta} = \left[T, \dot{T}, t^H, Ip, vi, ti, \eta, \tau, P_i, P_f, tr\right] \quad (1)$$

The $\boldsymbol{\theta}$ vector delivers orders to all the modules in MAOSIC for the given project, and it also guarantees the repeatability for the typical task. The materials, like reagents, are also parametrized to make the whole system digitalized. We define R_i the liquid reagent, as $\mathrm{R}_i = [i, v_i]$. R_i^s the solid reagent, as $\mathrm{R}_i^s = [i\ , w_i]$. Here v is the volume of liquid reagent, w is the weight of solid reagent, *i* is the serial number of the reagent in database. MAOSIC conduct the experimental operation by combining the parameter spaces of modules and the chemical reagent, and an operator is essential to connect the two parameters space. As shown in Supplementary Table 2, there are four well-defined operator symbols with the representation and examples, and each operator correlates the symbolic calculations within the module and chemical parameters. For example, $\mathrm{R}_1 \cdot SP_1$ can be interpreted as MAOSIC transfer the liquid chemical R_1 by the first Syringe pump, as indexed SP_1 from Supplementary Table 1.

Supplementary Table 2. Meaning and usage of operators.

Operator symbol	Representation	Usage example

$\cdot$	Reagent transfer	$\mathrm{R}_1 \cdot SP_1$ (transfer R_1 with SP_1)
$+$	Fusion of reagent	$\mathrm{R}_1 + R_2$ (Adding R_2 into R_1)
$\times$	React under environment	$\mathrm{R}_1 \times H$ (R_1 react under heating module)
$\vert$	Characterization	$ABS - CD \vert \mathrm{R}_1 \times A$ (Take ABS-CD measurement under heating module)

Supplementary Method 2:

Here, we tested the performance of different networks applying for the cloud lab (Shown in Supplementary Figure 3 and Supplementary Table 3). Under 5G network (provided by China Unicom & Ericsson), 405Mbps data rate and 19ms Ping significantly enhanced the preciseness of remote control of experimental platform.

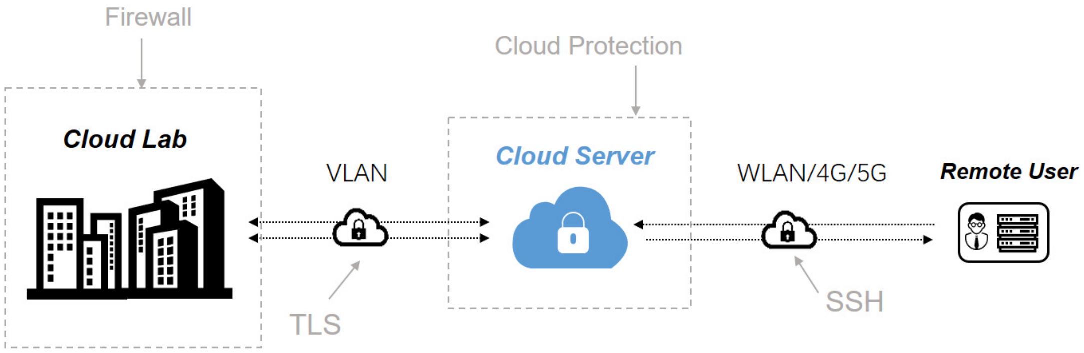

Supplementary Figure 3. Communication scheme of the system

Supplementary Table 3. Summary of tested Ping value and bit rate through different mobile networks

	WLAN	4G	5G
**Ping**	39 ms	75ms	19ms
**Transmission rate**	140Mbps	88Mbps	405 Mbps

Supplementary Method 3.

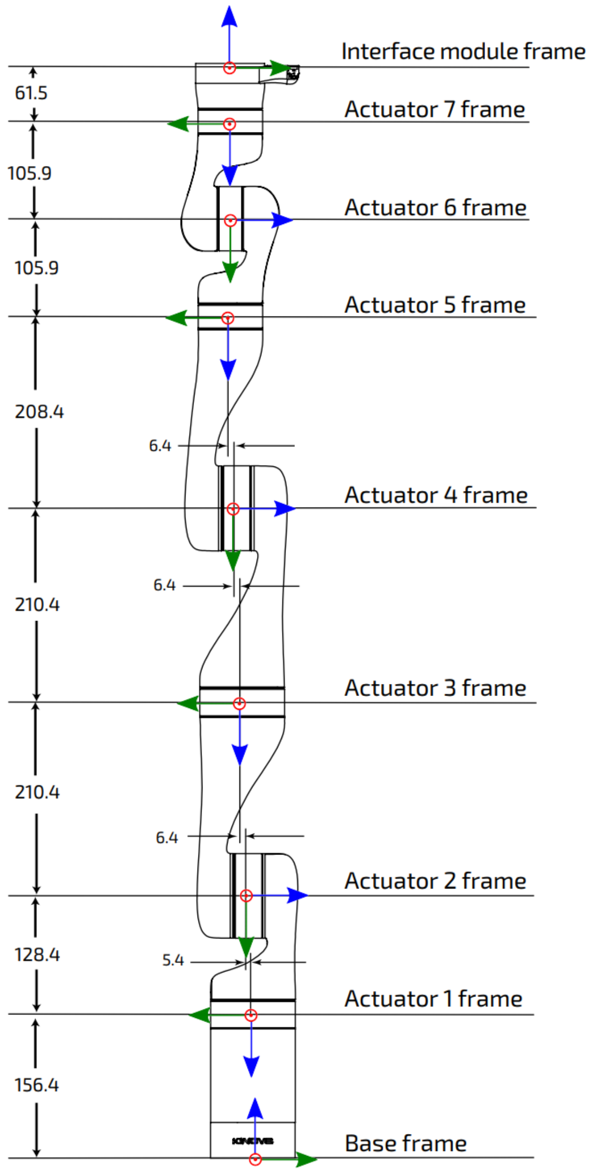

Supplementary Figure 4. Scheme of the robot arm (Gen 3, Kinova inc, Canada). 7 DoF (degree of freedom) spherical wrist frames definition and dimensions.

Supplementary Figure 4 defines reference frames for the base, joints (when all joint angles = 0) and end effector. Each frame is defined in terms of the previous frame via a transformation matrix. The diagram also indicates the link lengths and lateral offset values (measurements in mm). The maximum reach of the robot, as defined by the distance from the shoulder (actuator 2 frame) to the

interface module frame, is 90.2 cm.

Supplementary Table 4. Key parameters of Kinova Gen3 7 DoF robot arm

**Parameter**	**Values**
Weight	8.2kg
Payload	4 kg (mid-range continuous)
	4.5 kg (full-reach peak/temporary)
	1.1 kg (full-reach continuous)
Maximum reach (fully extended)	902 mm (7 DoF)
Degrees of freedom	7 DoF
Actuators	qty 3 (small)
	qty 4 (large)
Wrist interaction buttons	qty 2 (user-configurable; default for null space and Cartesian admittance control)
Power supply voltage	24 VDC (nominal, 18 to 30V)
Materials	Carbon fiber shell
	Aluminum
Communications and control	100 Mbps Ethernet for real-time 1 KHz control

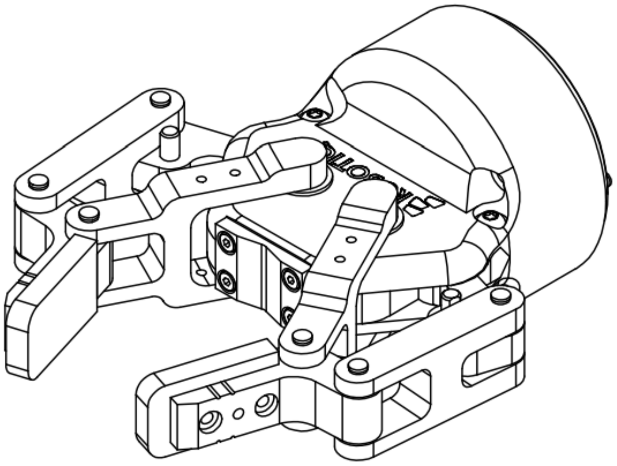

Supplementary Figure 5. Scheme of the gripper (2F-85, ROBOTIQ, Canada) integrated at the end of Kinova robot arm

Supplementary Table 5. Key parameters of ROBOTIQ gripper

Stroke	*85mm*
Grip Force	*20 to 235N*
Form-fit Grip Payload	*5 kg*
Friction Grip Payload	*5 kg*
Gripper Weight	*0.9 kg*
Closing speed	*20 to 150 mm/s*
Ingress protection (IP) rating	*IP40*

Automated microfluidic synthesis system and in-situ characterization tools

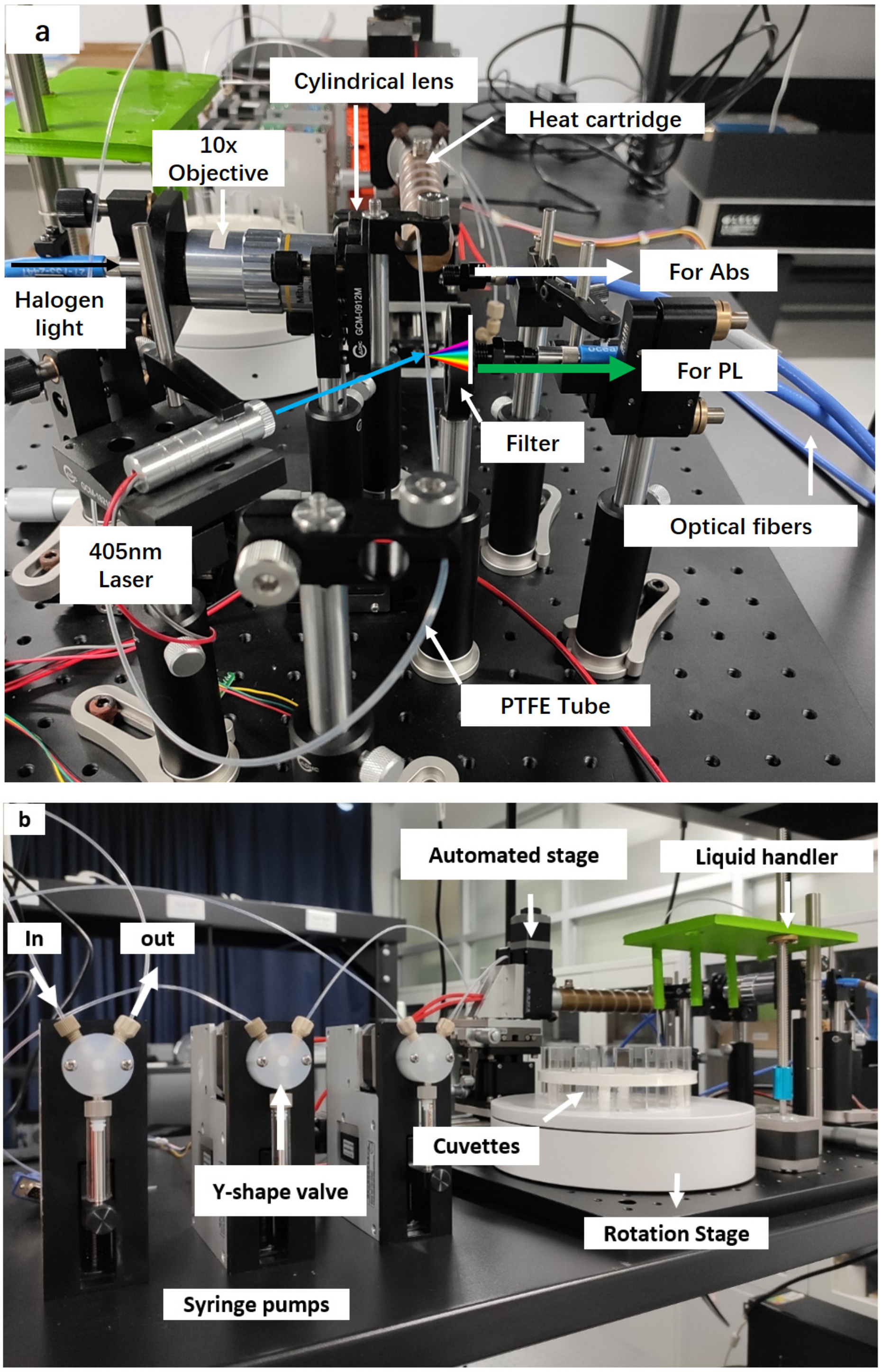

Supplementary Figure 6. Photograph of automated flow synthesis system.

Temperature calibration: Temperature calibration of chemical flow in heat zone was designed according to Kovalenko and deMello 's work in 2016 *[1]*. It was based on the decrease of fluorescence intensity of

Rhodamine B continuously with increasing the temperature [2]. The PL spectrum of Rhodamine B in water (100 μM) at a different position in optimizes heating zone (corresponding to different heating time) was collected in Supplementary Figure 7. PL intensity with under 1% variation among three-time points (1s, 5s, 25s), which quantitatively verified the fast heating efficiency of this heating module.

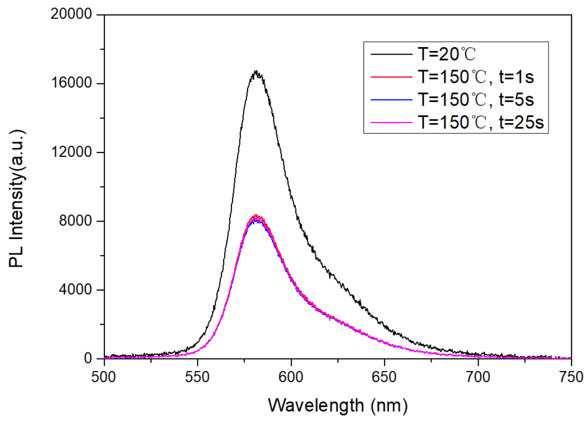

Supplementary Figure 7. PL spectrum of 100 μM Rhodamine B at 25 °C and 150 °C for temperature calibration. The red, blue and pink line represents the PL spectrum of Rhodamine B solution entering the Heating module at 1s, 5s, and 25s, separately. The nearly same PL intensity proved the fast heating ability of reaction flow within 1 second.

Liquid transfer module: A transfer module was designed as a connection center for different synthesis and characterization modules (Supplementary Figure 8). An up-down system made up with a stepping motor and screw rod was assembled for guiding the tube in-out of the solution container. The tube guider was manufactured by 3D printer with PLA materials.

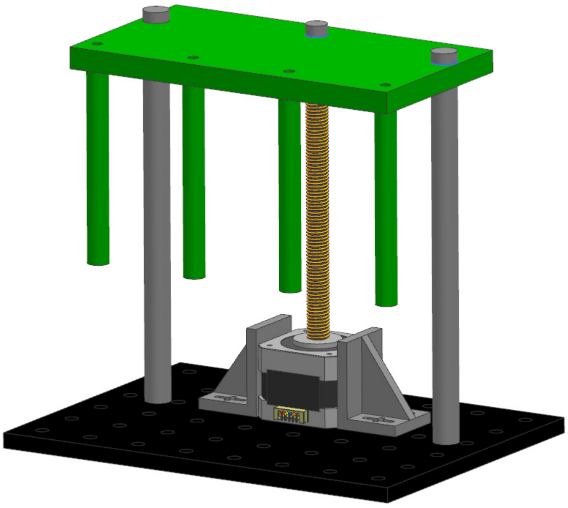

Supplementary Figure 8. Photograph and CAD model of the Liquid transfer module. The up-down system enables a speed range from 1mm/s to 5mm/s. The 3D-printed tube guide was designed to collect different solution container and microfluidics.

Supplemenatry Method 4.

Algorithms: The parameters for the SNOBFIT based reinforcement learning (RF) in this work is of the form like $(\mathcal{S}, \mathcal{A}, [\mathcal{P}_{as}], \mathcal{R})$. Here $\mathcal{S}$ denotes the set of state s, which is the set of all reaction parameter and an experimental result that has been experimented. State at timestamp i is denoted by $s_i = [a_0, r_0, \dots, a_i, r_i]$; $\mathcal{A}$ denotes the set of action a_i. In the context of reaction optimization. $\mathcal{A}$ is the combinations of reaction parameters such as reaction time and reactant concentration. Action at timestamp i is denoted by $a_i = [a_i^1, \dots, a_i^j, \dots, a_i^n]$; $[\mathcal{P}_{as}]$ denotes the state transition probability, here p_{as} decides what action a to make under the experimental condition s. This action decision policy is denoted by π; $\mathcal{R}$ denotes the reward function under state $\mathcal{S}$ and action $\mathcal{A}$. In the environment of reaction optimization, the reward at timestamp i is denoted by $r_i = [r_i^1, \dots, r_i^j, \dots, r_i^n]$, the reward function denotes the mapping $r_i = R(a_i)$. At a specific experimental condition a_i is mapped to get the difference between the experimental and the target then get the reward r_i.

For a timestamp i with the previous state s_{i-1}, the policy function decides the action to make by $a_i = \pi(s_{i-1})$. Then, continue the experiment with a_i, and get the reward $r_i = R(a_i)$. For the MAOSIC, architecture, the state could be updated by $s_i = s_{i-1} \cup [a_i, r_i]$. Policy function π performs local optimizations around the best conditions while searching for unexplored regions to ensure global optimality. For local optimization, the action is decided by searching from the best condition with a full quadratic model. For example, an action could be decided by approximately minimizing the local quadratic model. $q(a) = r_{best} + g^T(a - a_{best}) + \frac{1}{2}(a - a_{best})^T G(a - a_{best})$, where g and G are the gradients and the symmetric matrix estimated, respectively. [3]

For the global optimization, the action is decided by searching simultaneously in several promising sub-regions. For example, the parameter space is partitioned into sub-boxes each contains a history state [3]. The actions are chosen by

$$a = \begin{cases} \frac{1}{2}\left(\underline{a_i} + a_i\right), if \;\; a_i - \underline{a_i} > \overline{a_i} - a_i \\ \frac{1}{2}(\overline{a_i} + a_i), otherwise \end{cases}$$

, where $[\underline{a_i}, \overline{a_i}]$ is one of the largest sub-boxes of the current partition with history action a_i inside. The actions selected will finally be rounded into the nearest integral multiple of the resolution vector Δa, which is decided by the physics condition of reaction

parameters (here $\Delta a = (\Delta T_i, \Delta c) = (0.5℃, 0.01mmol/mL)$, T_i and c are the heating temperature and reactant concentration respectively). We can make a specific reaction parameter Δa^j large enough, then the local optimization would have little influence on this dimension because of the round procedure. For global search mentioned above, new actions would be selected in the most massive sub boxes.

Pseudocode for chiral $CsPbBr_3$ nanocrystals discovery

```
def HANDLE_REQUEST(request_id):
        target, reaction_id = FETCH_REQUEST_INFO(request_id)
        config = FETCH _REACTION_CONFIG(reaction_id)
        if not CHECK_RESOURCES(reaction_id):
                raise Exception
        reaction = GET_REACTION(config)
        optimizer = GET_OPTIMIZER(config)
        reaction.initialize()
        optimizer.initialize()
        LOAD_REACTION_HISTORY(optimizer, reaction_id)
        if OPTIMAL (optimizer.get_best())
                return optimizer.get_best()
        n_call = 0
        while n_call < config.max_call:
                action = optimizer.choose_action()
                result = reaction.experiment(action)
                reward = reaction.analyzer.calc_reward(result, target)
                STORE_RESULT(action, result)
                optimizer.update_env(action, reward)
                n_call = n_call + LENGTH(action)
                if OPTIMAL(optimizer.get_best()):
                        return optimizer.get_best()
        return optimizer.get_best()
```

Notes:

FETCH_REQUEST_INFO:

load request information from the database.

FETCH _REACTION_CONFIG:

loads the configure file of the reaction from the database, which contains all the information about how to synthesis and the hyper-parameter of the optimizer.

CHECK_RESOURCES :

checks if the hardware works well and the reagent is enough.

The reaction in charge of communicating with the hardware control interface and controlling the experiment optimizer choose the reaction parameters (action) and learn from the analysis result (reward).

LOAD_REACTION_HISTORY:

load all experiments about this action and update the state of the optimizer.

STORE_RESULT:

store the experiment results in the database.

Compile in cloud server: An example of compiling the input experimental formula to machine instruction code is shown in Supplementary Figure 9. This example shows how MAOSIC extracts information from input formula, and set up all experimental parameters in configuration file for $CsPbBr_3$ nanocrystals synthesis. The configuration file was generated through designed template. It decides the all adjustable or constant parameters for optimizing the experiment.

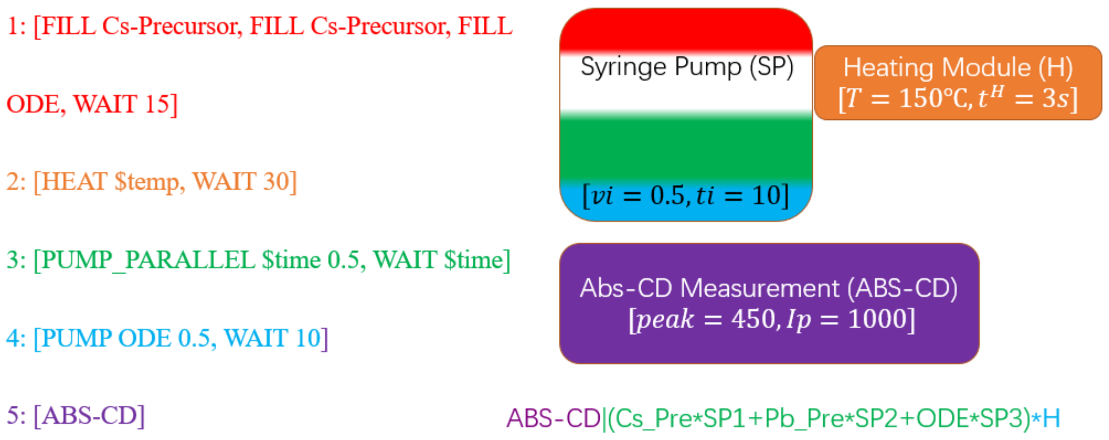

Supplementary Figure 9. Scheme of compiling process in MAOSIC. The color if icons and cods indicate different steps in experiment. Firstly, the formula (on right) with vectors of module parameters (in each icon) were input to MAOSIC. Then, the instruction codes (on left) were generated through compiling process.

Configuration file

```
{“type”: “fluid reaction”,
“name”: “CsPbBr3 Nanocrstals”,
“reagent”: [{“formula”: “Cs-Precursor”, “ratio”: 1},
{“formula”: “Pb-Precursor”, “ratio”: 2},
{“formula”: “ODE”, “ratio”: 1}],
“metric”: ('CD', [“wavelength”, “peak”]),
“param”: {“temp”: {“default”: 100, “bound”: [90, 180], “dx”: 1, “var”: 1},
“ODE”: {“default”: 1, “bound”: [0.01, 100.0], “dx”: 0, “var”: 1},
“time”: {“default”: 20, “bound”: [20, 20], “dx”: 0, “var”: 0}},
“init”: [“PUMP_INIT”],
“experiment”: ['FILL Cs-Precursor', 'FILL Pb-Precursor', 'FILL ODE', 'WAIT 15', 'HEAT $temp',
'WAIT 30', 'PUMP_PARALLEL $time 0.5', 'WAIT $time', 'PUMP ODE 0.5', 'WAIT 10', 'ABS-CD'],
“optimizer”: “snobfit”,
“max_expts”: 250,
“nreq”: 0}
```

Supplementary Figure 10. An example configure file for chiral $CsPbBr_3$ nanocrystals synthesis.

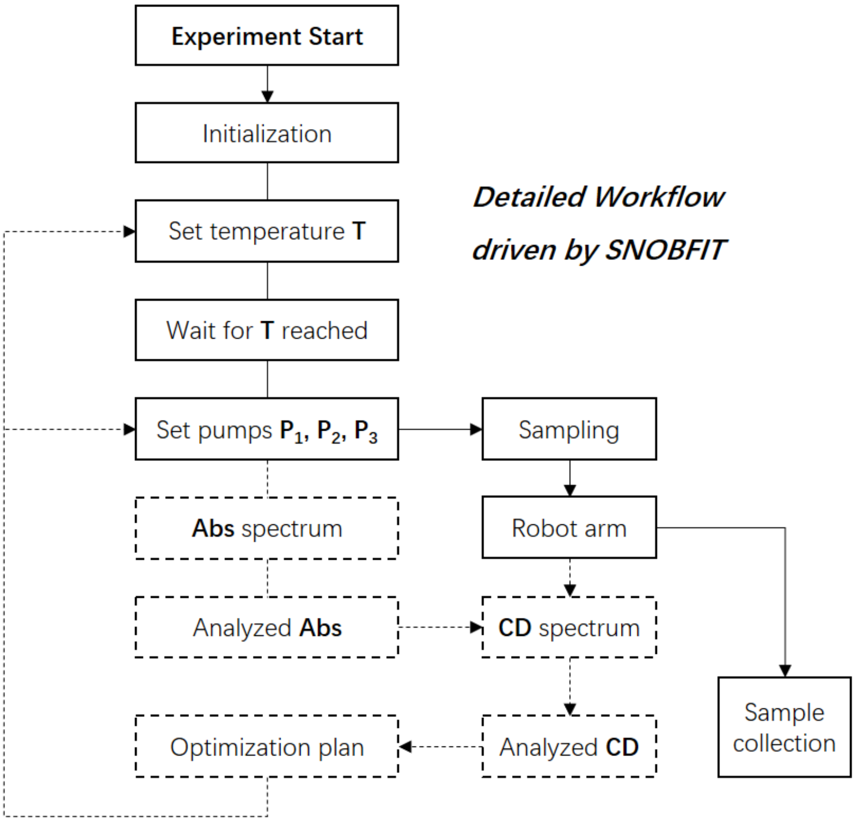

Supplementary Figure 11. Workflow of the experiment driven by MAOSIC. **T** indicates the reaction temperature. **P1, P2, P3** refer to the injection rate of each syringe pumps, which controlled the reaction time and precursors concentration.

Supplementary Method 5

Morphology and temperature dependence of the screw dislocations: Besides the chiral dichroism of the nanomaterials induced by the screw dislocations, the possibility of forming such a dislocation with respect to different morphologies and temperature also requires attention. It is hereby analysed by comparing the energy resulted from the screw dislocation and the thermal fluctuation of the temperature, by adopting the most common expression $E_{thermal} = \frac{3}{2} n k_B T$.

As for the energy induced by the screw dislocation, it can be calculated from the distortion of geometry of the nanomaterials and the stress induced. The energy has already been defined as the energy density per unit length as

$$e_{stress} = \frac{\mu b^2}{4\pi}\left(ln\left(\frac{r_{outer}}{r_0}\right) - 1\right), \tag{2}$$

where μ is the shear modulus, b is the magnitude of the burgers vector, r_{outer} is the outer most range of the dislocation on the radial direction of the cylinder (not the radius of the cylinder), and r_0 (r_0 is taken to be approximately b) and r_{outer} gives the bounded region of the screw dislocation[4]. So, this energy is linear with the length on the vertical direction, where the screw dislocation occurs. If it is assumed that the screw dislocation occurs only for a finite length l, the energy would be $e_{stress} l$.

As for the thermal fluctuation,

$$E_{thermal} = \frac{3}{2} n k_B T = \frac{3}{2}\frac{\rho \pi R^2 L}{M} k_B T. \tag{3}$$

By comparing the magnitude of $E_{thermal}$ and $E_{dis} = e_{stress} l$ per site, it could be observed that: as the radius growing, i.e. the morphology approaches 2-D, the energy is at first very small because of the size, the energy of dislocation then increases but falls below the temperature effect afterwards, which means the defected morphology is more likely to occur at if the morphology is more similar to 2-D; as for the temperature, the energy of dislocation also falls below the thermal energy after reaching some threshold, indicating that the defects are more likely to occur at higher temperature.

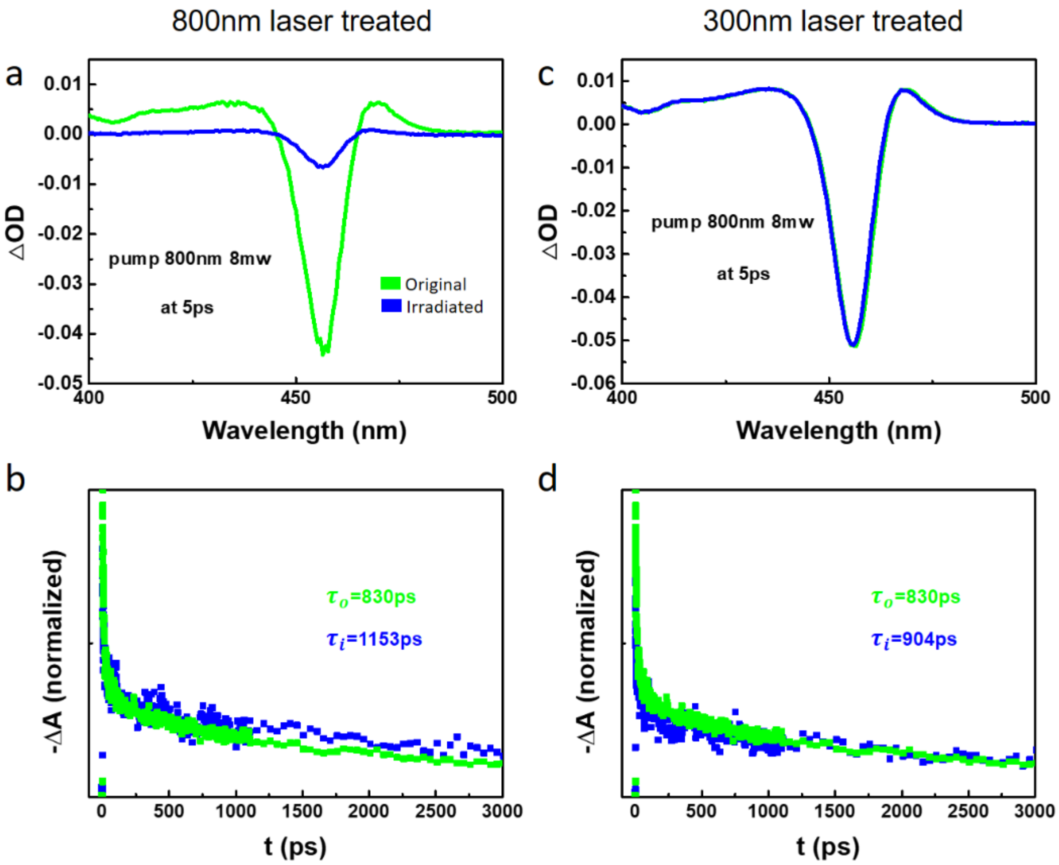

Supplementary Figure 12. Transient absorption spectrum (TAS) at 5ps (ΔOD means the difference of optical density before and after the optical excitation of sample) and fitting results of average carries lifetime (ΔA means the difference of absorption) for global optimal sample (a) (b) after 800nm laser and (c) (d) 300nm laser irradiated. All characterization was taken under 800nm pump laser with 8mW power.

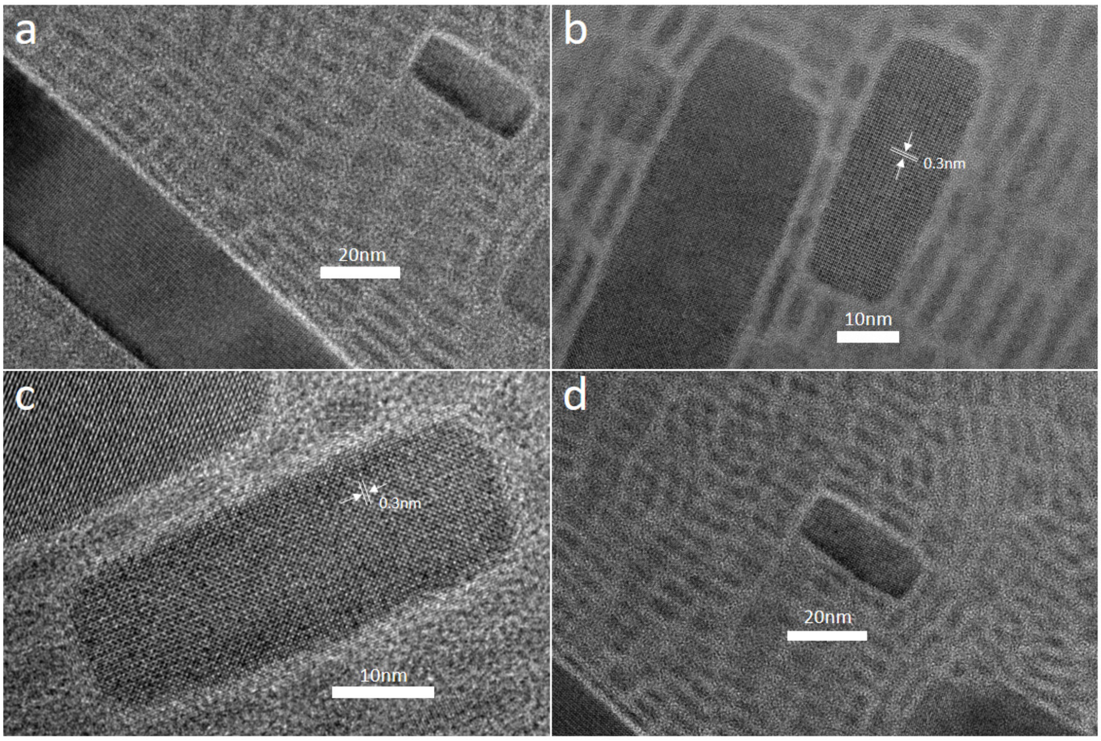

Supplementary Figure 13. TEM images of aggregation among $CsPbBr_3$ nanocrystals.

Supplementary Discussion 1.

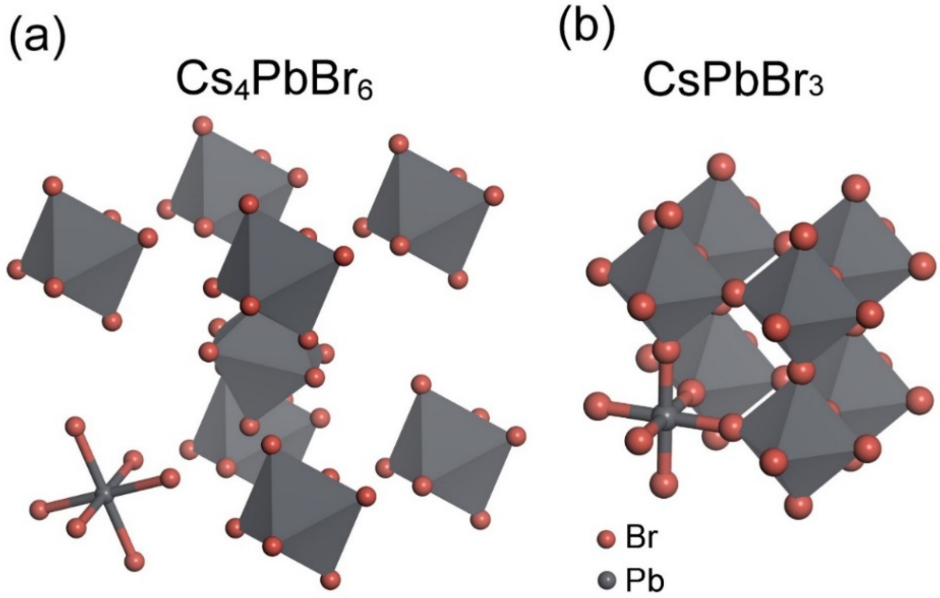

Supplementary Figure 14. Crystal structure for (a) Cs_4PbBr_6 and (b) cubic $CsPbBr_3$. The brown and gray color represents the Br and Pb respectively. The Cs atoms are not shown for clarity.

Phase transition prediction: Supplementary Figure 14 shows the crystal structure and local chemical bond information for Cs_4PbBr_6 and cubic CsPbBr3 respectively. The main difference between the two phases is that the $PbBr_6$ octahedrons share vertexes in CsPbBr3 and the same $PbBr_6$ octahedrons are isolated in Cs_4PbBr_6. For the phase transition from $CsPbBr_3$ to Cs_4PbBr_6, some Pb-Br bonds have to be broken in the $CsPbBr_3$ crystal phase to from the isolated $PbBr_6$ octahedron, and more Br- ions are essential for the stoichiometry, thus the octahedrons in the edge of $CsPbBr_3$ structure are easier to form the $PbBr_6$ octahedron with the external Br- ions from environment. In order to confirm the energy scale for the Cs_4PbBr_6 phase to emerge, we apply Nudge Elastic Band (NEB) [5] calculations with eight transition state images for the energy barrier investigations. As shown in Supplementary Figure 15, here we use a Cs21Pb8Br37 nanocluster to mimic the locale phase transitions. To form an isolated PbBr6 octahedron, it needs one additional Pb-Br bond, which requires an additional Br- ion from external environment. As indicated by the red circle in structure '1' and '2', once the Br- ion approaches the Pb atoms, a newly-formed Pb-Br bond comes into being, as indicated by the red bond in structure '3', and a Pb-Br bond previous shared by the nearby octahedrons is broken, as shown by the blue color in the same structure. Eventually the Br- ion from environment comes into bonding and the

structure ‘4’ becomes more Cs_4PbBr_6 -like. The energy barrier for this processing is about 0.42 eV. We think the occurrence of Cs_4PbBr_6 phase is due to the event described in Supplementary Figure 15, where the energy barrier is overcoming by the irradiation or consequential thermal fluctuation.

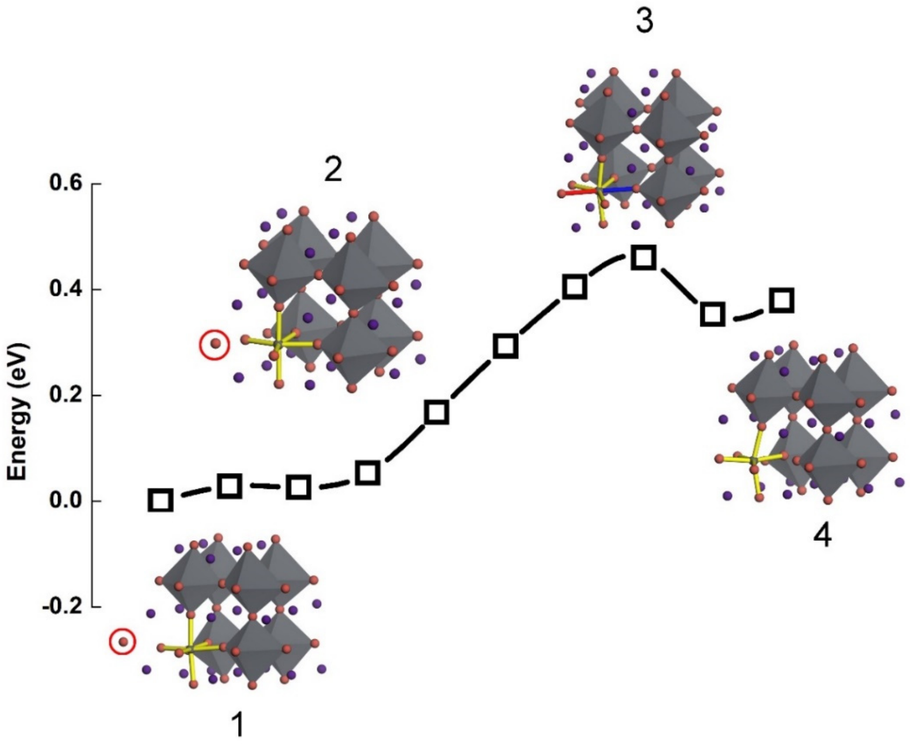

Supplementary Figure 15. NEB calculations for the phase transition from $CsPbBr_3$ to Cs_4PbBr_6. For simplicity, A $Cs_{21}Pb_8Br_{37}$ nanocluster is used for the simulation. The structure ‘1’ and ‘4’ represent the initial ($CsPbBr_3$) and final (Cs_4PbBr_6) state, ‘2’ and ‘3’ represent the middle stage structures. The red circle in structure ‘1’ and ‘2’ represent the Br- ion from environment. The red and blue bonds in structure ‘3’ indicates the newly-formed bond from Pb and external Br- ion and tentatively-broken Pb-Br bond. The energy barrier is about 0.42 eV.

Supplementary Method 6.

$$-\frac{\hbar^2}{2m}\left[\frac{1}{r}\frac{\partial}{\partial r}\left(r\frac{\partial}{\partial r}\right)+\frac{1}{r^2}\frac{\partial^2}{\partial\varphi^2}+\frac{\partial^2}{\partial z^2}+\frac{2\beta}{r^2}\frac{\partial}{\partial\varphi}\frac{\partial}{\partial z}-\frac{2\beta^2}{a^2r^2}+V(r,\varphi,z)\right]\psi+cr^2\psi=E\psi \quad (4)$$

The solution of the Schrodinger equation has the form $\psi = w(r)e^{i(l\varphi+kz)}$, where $l \in Z^*$ and k is the wave vector along the z direction, $k = 2\pi m / L, m \in Z^+$. Thus,

$$w''+\frac{1}{r}w'+\left[-\frac{A}{r^2}-k^2-\frac{2mc}{\hbar^2}r^2\right]w=-\frac{2mE}{\hbar^2}w \quad (5)$$

where $A = l^2 + 2\beta kl + \frac{2\beta^2}{a^2}$. In defining more variables as

$$\kappa=\frac{i}{4\sqrt{C}}\left(k^2-\frac{2mE}{\hbar^2}\right)=\frac{1}{4}\sqrt{\frac{\hbar^2}{2mc}}\left(k^2-\frac{2mE}{\hbar^2}\right),\mu=\frac{\sqrt{A}}{2},\rho=\sqrt{\frac{2mc}{\hbar^2}}r^2,$$

the differential equation could be further simplified, and a pair of linearly independent solutions at $\rho = 0$ is given by the Whittaker-M function[6]

$$M_{\kappa,\mu}(\rho)/r \text{ and } M_{\kappa,-\mu}(\rho)/r$$

Noting that $\mu > 1/2$ and the behavior of $M_{\kappa,\mu}(\rho)$ as $\rho \to 0$, $M_{\kappa,\mu}(\rho)$ remains bounded while $M_{\kappa,-\mu}(\rho)$ blows up at $\rho = 0$. Thus, $w(\rho) \propto M_{\kappa,\mu}(\rho)/r$, because the asymptotic expansion near 0 of the Whittaker function[7] is $M_{\kappa,-\mu}(\rho) \to \rho^{-\mu+\frac{1}{2}}(1 + O(\rho))$. According to Whittaker[6], the Whittaker-M function could be expressed also as a polynomial of the form (in *Chapter XVI* of the referenced book):

$$M_{\kappa,\mu}(\rho)=\rho^{\frac{1}{2}+\mu}e^{-\frac{1}{2}\rho}\left(1+\sum_{t=1}^{\infty}\frac{\prod_{p=1}^{t}\left(p-\frac{1}{2}+\mu-\kappa\right)}{\prod_{q=1}^{t}(2\mu+q)}\frac{\rho^t}{t!}\right). \quad (6)$$

So, to terminate the polynomial, the term must equals zero after some t, assume this $t_{max} = n$ as the quantum number ($n \in Z^+$), the energy of the corresponding quantum number could be defined as:

$$\mu-\kappa+n-\frac{1}{2}=0,$$

$$E_{nlk}=\frac{\hbar^2k^2}{2m}+2\hbar\sqrt{\frac{2c}{m}}\left(\frac{\sqrt{A}}{2}+n-\frac{1}{2}\right)=\frac{\hbar^2k^2}{2m}+2\hbar\sqrt{\frac{2c}{m}}\left(\frac{\sqrt{l^2+2\beta kl+\frac{2\beta^2}{a^2}}}{2}+n-\frac{1}{2}\right). \quad (7)$$

Thus, by defining the normalization factor N_{nlk}, and keeping the expressions above,

$$\psi_{nlk}(r,\varphi,z)=N_{nlk}WhittakerM\left(\frac{1}{4}\sqrt{\frac{\hbar^2}{2mc}}\left(k^2-\frac{2mE_{nlk}}{\hbar^2}\right),\frac{\sqrt{l^2+2\beta kl+\frac{2\beta^2}{a^2}}}{2},\sqrt{\frac{2mc}{\hbar^2}}r^2\right)r^{-1}e^{i(l\varphi+kz)}. \quad (8)$$

Supplementary Method 7.

Assume the incident light propagates along the z-axis with a wave vector of magnitude $q = \sqrt{\epsilon}\omega/c$, the interaction of the light and the material with right-hand polarization $(+)$ and left-hand polarization $(-)$ could be described by the Hamiltonian[8]

$$H_{\pm} = \mp i \frac{A_0 e}{\sqrt{2}mc} e^{iqz} p_{\pm 1} \tag{9}$$

where A_0 is the magnitude of the vector potential, $-e$ is the electron charge, c is the speed of light in vacuum. The covariant cyclic components of the momentum operator are given by

$$p_{\pm 1} = \hbar \frac{e^{\mp i\varphi}}{\sqrt{2}} \left(\frac{1}{r}\frac{\partial}{\partial \varphi} \pm i \frac{\partial}{\partial r} \right) \tag{10}$$

Assume also that the electrons obey the Fermi-Dirac distribution $f_{nl}(k) = 2\left(e^{\frac{E_{nl}(k) - E_{nl}(\kappa_{nl}) - \mu}{k_B T}} + 1 \right)^{-1}$, where μ is the effective chemical potential, and κ_{nl} is the value of k at which the minimum energy is achieved for the subband (n, l). Thus, the intraband absorption in total is to be calculated by summing over all possible excitations whose energy gap equals the energy of the incident light:

$$\Gamma(\omega) \propto \int dk \int dk' \sum_{\pm l} \sum_{n'l'} f_{nl}(k) \left| \langle n'l'k' | H_{\pm} | nlk \rangle \right|^2 \delta\left(\frac{E_{n'l'}(k') - E_{nl}(k)}{\hbar} - \omega \right). \tag{11}$$

By taking into consideration only the excitation of the ground state, the calculation result showed the Cotton effect[9]. The sign of the Cotton effect was found to be relevant to the screw dislocation: a positive β will lead to a negative Cotton effect while a negative β will result in a positive Cotton effect. Thus, the reverse of the circular dichroism observed in experiments must be related to the alteration of the pattern of the nanoplates.

Supplementary Method 8.

The ligand-induced chirality is studied in a simplified model, where the surface effect is believed to be similar to the diffusion, which obeys the Fick's second law of diffusion. Since it alters not the electronic structure of the material, the Schrodinger equation method will not apply. The diffusion is applied here since, according to DFT study[10], the chiral excitation comes from the excitation of electrons from the nanoplates to the chiral organics on the surface. Assume that the closer the electron is to the surface, i.e. the organics, the easier it will be excited, which means that the lifetime of that electron would be shorter. Assume further this lifetime is proportional to the distance of the electron to the surface, the continuity equation along the radial direction, from the Fick's second law, writes

$$D\frac{d^2n}{dr^2} = \frac{n_0}{\tau(R-r)}, \tag{12}$$

where D is the diffusion coefficient, n_0 is the initial concentration of electrons, $\tau(m^{-1}s)$ is the time coefficient to determine the lifetime. The solution is

$$n = \frac{n_0}{D\tau}\left(r\left(1 - ln\big(D\tau(R-r)\big)\right) + Rln\left(\frac{R-r}{R}\right)\right). \tag{13}$$

To take into account the temperature dependence, the parameter D is altered. According to the Einstein relation, $D = \mu k_B T$ is proportional to temperature. By putting in this information, the flux of the solution would decrease with temperature. This means the chirality will also decrease since the excited electrons are lessened. In such a case, the chirality induced by surface attachment of chiral organics also decays with temperature. But this mechanism introduces no reversal of the circular dichroism. Due to the reversal observed in our experiments, it can be concluded that the chirality in our experiments was not generated by the surface attachment of ligands.

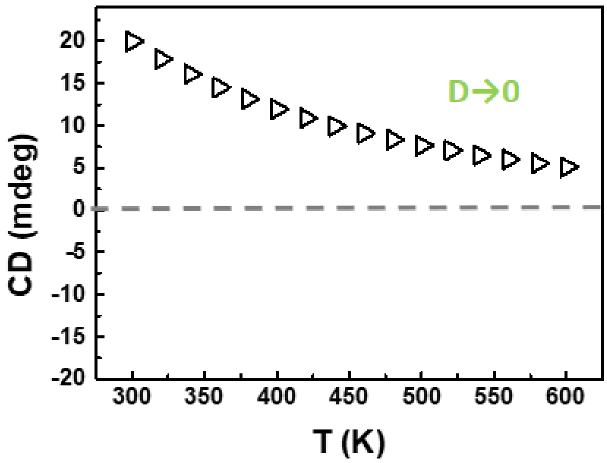

Supplementary Figure 16. Calculation of temperature-dependent CD induced by surface chiral ligands.

Supplementary Reference

[1] I. Lignos, S. Stavrakis, G. Nedelcu, L. Protesescu, A. J. deMello, M. V. Kovalenko, *Nano Lett* **2016**, *16*, 1869-1877.

[2] R. M. Guijt, A. Dodge, G. W. K. van Dedem, N. F. de Rooij, E. Verpoorte, *Lab on a Chip* **2003**, *3*, 1-4.

[3] W. Huyer, A. Neumaier, *ACM Trans. Math. Softw.* **2008**, *35*, 1-25.

[4] J. P. Hirth, J. Lothe, T. Mura, *Journal of Applied Mechanics* **1983**, *50*, 476.

[5] H. JÓNSSON, G. MILLS, K. W. JACOBSEN, in *Classical and Quantum Dynamics in Condensed Phase Simulations*, pp. 385-404.

[6] E. T. Whittaker, G. N. Watson, *A course of modern analysis*, Cambridge university press, **1996**.

[7] F. W. Olver, D. W. Lozier, R. F. Boisvert, C. W. Clark, *NIST handbook of mathematical functions hardback and CD-ROM*, Cambridge university press, **2010**.

[8] aG. H. Wagnière, G. L. Rikken, *Chemical Physics Letters* **2009**, *481*, 166-168; bA. S. Baimuratov, I. D. Rukhlenko, Y. K. Gun'ko, A. V. Baranov, A. V. Fedorov, *Nano letters* **2015**, *15*, 1710-1715.

[9] T. Lowry, *Nature* **1933**, *132*, 552-552.

[10] J. Cheng, J. Hao, H. Liu, J. Li, J. Li, X. Zhu, X. Lin, K. Wang, T. He, *ACS Nano* **2018**, *12*, 5341-5350.
